# Decreased sensitivity to 1,25-dihydroxyvitamin D3 in T cells from the rheumatoid joint

**DOI:** 10.1016/j.jaut.2017.10.001

**Published:** 2018-03

**Authors:** Louisa E. Jeffery, Peter Henley, Nefisa Marium, Andrew Filer, David M. Sansom, Martin Hewison, Karim Raza

**Affiliations:** a Institute of Metabolism and Systems Research, University of Birmingham, Birmingham, B15 2TT, UK; b Institute of Immunology and Immunotherapy, University of Birmingham, Birmingham, B15 2TT, UK; c Institute of Inflammation and Ageing, University of Birmingham, Birmingham, B15 2TT, UK; d University Hospitals Birmingham NHS Foundation Trust, Birmingham, B15 2WB, UK; e UCL Institute of Immunity and Transplantation, University College London UK; f Centre for Endocrinology, Diabetes and Metabolism, Birmingham Health Partners, Birmingham, B15 2TT, UK; g Department of Rheumatology, Sandwell and West Birmingham Hospitals NHS Trust, Birmingham, UK

**Keywords:** Vitamin D, Rheumatoid arthritis, Synovial fluid, Peripheral blood, T cell, Vitamin D receptor

## Abstract

1,25-dihydroxyvitaminD_3_ (1,25(OH)_2_D_3_), has potent anti-inflammatory effects, including suppression of IL-17 + and IFNγ+ T cells implicated in rheumatoid arthritis (RA), but efficacy at the site of active disease is unclear. To investigate this, T cells from synovial fluid (SF) and paired blood of patients with active RA were studied. 1,25(OH)_2_D_3_ had significantly less suppressive effect on Th17 cells (IL-17+IFNγ-) and Th17.1 cells (IL-17+IFNγ+) from SF compared to those from blood, and had no effect on SF CD4^+^ or CD8^+^ IFNγ+ T cell frequencies. Memory T cells (CD45RO+) predominate in SF, and 1,25(OH)_2_D_3_ had less effect on memory T cells relative to naïve (CD45RA+) T cells. RT-PCR and flow cytometry showed that this was not due to decreased expression of the vitamin D receptor or its transcription partners in memory T cells. Further studies using stimulated CD4^+^ T cells sorted according to IL-17 and IFNγ expression confirmed the ability of 1,25(OH)_2_D_3_ to suppress pre-existing cytokines. However, 1,25(OH)_2_D_3_ was most effective at suppressing *de novo* IL-17 and IFNγ induction. Correspondingly, T cell responses to 1,25(OH)_2_D_3_ correlated directly with capacity for phenotype change, which was lower in cells from SF compared to blood. These findings indicate that anti-inflammatory effects of 1,25(OH)_2_D_3_ in active RA are impaired because of reduced effects on phenotype-committed, inflammatory memory T cells that are enriched in SF. Restoration of 1,25(OH)_2_D_3_ responses in memory T cells may provide a new strategy for treatment of inflammatory diseases such as RA.

## Highlights

• T cells from rheumatoid arthritis (RA) synovial fluid are less sensitive to vitamin D than T cells from blood.
• Memory T cells that predominate in synovial fluid of RA joints are less sensitive to vitamin D than naïve T cells.
• Memory T cells from synovial fluid show strong phenotype commitment.
• Vitamin D is unable to exert anti-inflammatory effects on phenotype committed T cells.
• Vitamin D exerts anti-inflammatory effects by suppressing *de novo* synthesis of T cell cytokines.

## Graphical abstract

**Figure:**
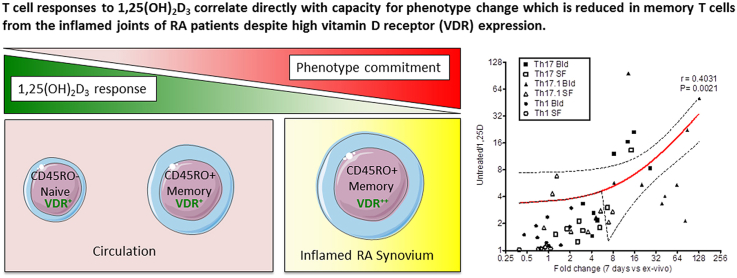

## 1 Introduction

The active form of vitamin D, 1,25-dihydroxyvitamin D_3_ (1,25(OH)_2_D_3_) promotes anti-inflammatory responses in a diverse array of cell types, supporting the potential use of vitamin D in the prevention and/or treatment of inflammatory disorders [1], [2]. In previous studies we have shown that anti-inflammatory actions of vitamin D may occur indirectly through localized synthesis of 1,25(OH)_2_D_3_, and reduced expression of major histocompatibility and co-stimulatory molecules by antigen-presenting dendritic cells (DCs), monocytes and macrophages [3], [4], [5], [6]. However, 1,25(OH)_2_D_3_ can also act directly on T-lymphocytes (T cells), inhibiting their proliferation [7], especially under conditions of weak co-stimulation [8], and suppressing their production of pro-inflammatory cytokines such as IFNγ, IL-17 and IL-21 [9], [10], [11], [12], [13], whilst promoting their expression of regulatory markers including CTLA-4, FoxP3, and IL-10 [13] even in the presence of pro-inflammatory cytokines [14]. Crucially almost all of these observations have stemmed from experiments using T cells from the blood of healthy donors and much less is known about the effects of 1,25(OH)_2_D_3_ in established inflammatory disease, especially its effects upon T cells from inflamed compartments such as the joint of a patient with rheumatoid arthritis (RA).

Epidemiology suggests that many autoimmune diseases and common chronic inflammatory diseases such as RA are associated with vitamin D-deficiency [15], [16], [17]. Vitamin D metabolites such as 1,25(OH)_2_D_3_ may therefore provide an alternative strategy for the prevention and/or treatment of RA [18], possibly as an adjunct to existing RA therapies [8]. Previous studies have highlighted aberrant metabolism of vitamin D in disease-affected synovial fluid (SF) from RA patients [19], but the impact of 1,25(OH)_2_D_3_ on T cells from the site of inflammation, the inflamed joint, has yet to be studied.

IFNγ+ Th1 cells and IL-17 + Th17 cells are regarded as important mediators of chronic synovial inflammation. Increased levels of their hallmark and differentiating cytokines are found in the serum and synovial fluid of patients relative to controls [20], and elevated frequencies of both have been detected in the blood and joints of RA patients [21]. IFNγ produced by Th1 cells promotes APC maturation whilst IL-17 has pleiotropic effects, driving fibroblast-like synoviocytes (FLS) to release pro-inflammatory cytokines and chemokines [22], that further amplify the inflammation by facilitating the recruitment and retention of immune cells, including CCR5+CXCR3+ Th1 cells [23], [24] and CCR6+ Th17 [25], [26] cells into the joint. IL-17 from Th17 cells also promotes cartilage and bone resorption by stimulating MMP release from FLS [22] and the induction of RANKL on FLS and osteoblasts leading to activation of RANK + osteoclasts [27]. The ability of 1,25(OH)_2_D_3_ to affect T cell function is important in RA, as T cells accumulate in the RA synovium and genetic risk factors for RA are largely related to T cell activation [20], [21], [22], [23], [24], [25]. The aim of the current study was to investigate further the potential use of vitamin D as a therapy for RA, by determining whether anti-inflammatory effects of 1,25(OH)_2_D_3_ are achievable on T cells from the site of inflammation.

## 2 Materials and methods

Patients were recruited for the study if they fulfilled 1987 ACR criteria for RA [28]. All patients and age and gender-matched healthy controls gave full written consent. Patient demographics are summarized in Table 1. Ethical approval for the work was granted by Solihull Research Ethics Committee (REC reference number 07/Q2706/2) and the University of Birmingham Ethics Committee (ERN_14-0446). For naïve and memory T cell comparison studies, as well for as cytokine-expression cell capture experiments, cells were isolated from fully anonymysed leukocyte cones obtained from the National Blood Service, Birmingham, UK.

**Table 1:** **Patient Demographics**: Disease activity score 28 based upon C Reactive Protein (CRP) (DAS28 (CRP); Erythrocyte sedimentation rate (ESR); Rheumatoid Factor (RF); anti-cyclic citrullinated peptide antibody (anti-CCP); conventional synthetic and biological disease modifying anti-rheumatic drugs (csDMARDs/bDMARDs).

Patient	Gender	Age (yrs)	Disease duration (yrs)	DAS28 (CRP)	CRP (mg/dl)	ESR (mm/hr)	RF (±)	Anti-CCP (±)	cs/DMARDs/bDMARDs
1	f	65	4	2.89	<5	37	+	–	methotrexate, rituximab
2	m	85	4	5.18	138	NA	–	+	Sulfasalazine, prednisolone
3	m	65	3	4.88	81	72	+	+	nil
4	f	65	4	4.84	10	41	+	–	methotrexate, rituximab
5	m	42	6	5.30	95	27	–	NA	sulfasalazine, methotrexate, prednisolone, tocilizumab
6	f	40	<1	3.18	14	26	–	–	methotrexate
7	m	47	2	5.48	34	NA	+	NA	methotrexate
8	m	42	6	4.19	23	7	+	+	methotrexate, prednisolone, rituximab
9	f	40	<1	4.73	59	57	–	–	sulfasalazine
10	m	42	6	4.82	14	2	–	NA	sulfasalazine, methotrexate, prednisolone, tocilizumab
11	m	45	4	8.21	166	130	–	–	nil
12	m	59	3	6.21	29	26	+	+	methotrexate
13	f	55	5	5.08	19	42	+	–	methotrexate, sulfasalazine
14	f	35	5	5.83	20	17	+	+	methotrexate
15	f	68	27	5.10	86	46	+	+	methotrexate, etanercept

### 2.1 Cell isolation and culture

Synovial Fluid (SF) was extracted by ultrasound guidance as described previously [29] or by palpation guidance. Prior to SF Mononuclear Cell (SFMC) isolation, SF was treated with hyaluronidase (10U/ml) for 30 min at 37 °C. PBMCs and SFMCs were isolated by the Ficoll-Paque PLUS method of density gradient centrifugation (GE Healthcare). SF was layered on Ficoll-Paque PLUS undiluted, fresh blood was diluted 1:1 with PBS and leukocyte cones were diluted 1:4 with PBS before layering. Isolated SFMCs and PBMCs were cultured at 37 °C, 5% CO_2_ in RPMI 1640 medium and supplemented with 1% penicillin and streptomycin, 2 mM l-glutamine, and 5% self-serum or SF that was pre-filtered through a 22 μm filter. For *ex vivo* cytokine expression analysis, cells were allowed to rest overnight at 1 × 10^6^ cells/ml without stimulation before being stimulated for 6–7 h with phorbol myristate acetate (PMA) (50 ng/ml) and ionomycin (1 μM). Brefeldin A (10 μg/ml) was added during the last 4–5 h. For stimulation mononuclear cells were treated with anti-CD3 (0.5 μg/ml, clone OKT3) at 2.5 × 10^5^ cells/ml. 1,25(OH)_2_D_3_ was added to cultures at 100 nM and ethanol used as a vehicle control at 0.1%. At seven days, cells were restimulated with PMA/ionomycin in the presence of brefeldin A for cytokine expression analysis by flow cytometry.

For experiments using isolated CD45RA + CD4^+^ naïve T cells, CD45RO + CD4^+^ memory T cells and CD14 ^+^ monocytes, cells were enriched by negative selection using cell separation reagents (StemCell Technologies and Biolegend). For 24 h post-stimulation analysis of gene expression, T cells were stimulated with anti-CD3/CD28 dynabeads (Life Technologies) at a ratio of 1 bead: 2 T cells in medium supplemented with 5% human AB serum (TCS Biosciences, Buckingham UK). For longer-term stimulations a ratio of 1 bead: 4 T cells was used. Where T cells were stimulated with monocytes, a ratio of 1 monocyte: 4 T cells and OKT3 0.5 μg/ml was used.

### 2.2 Isolation and culture of Th17, Th17.1 and Th1 cells

Expanded populations of Th17, Th17.1 and Th1 cells were generated by stimulating magnetically purified monocytes and CD4^+^ T cells at 1:5 ratio with 0.5 μg/ml antiCD3 for seven days. IL-17-PE and IFNγ-APC cytokine secretion detection kits (Miltenyi Biotech) were used to label live Th17, Th17.1 and Th1 cells. In brief, cultures were re-stimulated with Phorbol 12,13-dibutyrate (PDBu) (10 ng/ml) and ionomycin (1 nM) for 2 h before labeling with IL-17 and IFNγ catch reagents on ice at 10 × 10^6^ cells/80 μl MACS buffer for 5 mins. Cells were transferred to pre-warmed RPMI and incubated for 40 mins at 37 °C at 4 × 10^5^ cells/ml under continual rotation. Cells were then diluted 1:1 with ice-cold MACS buffer and chilled on ice for 10 min before centrifuging and labelling with IL-17-PE and CD3-PerCP for 15 min on ice with addition of IFNγ-APC during the final 10 min. After washing, Th17, Th17.1, Th1 and cytokine double-negative (DN) populations were collected into RPMI by FACS. Sorted T cells were then stimulated with negatively enriched (StemCell Technologies) and CD14^+^ FACS-purified allogenic monocytes at 1:4 ratio and 0.5 μg/ml anti-CD3 (OKT3) for 2 days in the presence of 40units/ml IL-2 (Immunotools) ± 100 nM 1,25(OH)_2_D_3_. Cell purities were >99% for Th17, Th1, DN and monocytes and >90% for Th17.1 cells.

### 2.3 Flow cytometry

CD45-RO + frequencies were assessed directly *ex vivo* by surface staining at 4 °C in PBS with antiCD45RO-FITC, CD3-PE and CD4-APC (all from BD Biosciences). For post-stimulation cultures, dead cells were labelled with near-IR LIVE/DEAD fixable dead cell stain (Molecular Probes, Life Technologies) before fixation. For analysis of regulatory markers: CTLA-4, Foxp3 and CD25, cells were fixed, permeabilised and stained with ebioscience/Thermofisher Foxp3 staining buffers according to the manufacturer's instructions. For analysis of cytokine expression, PMA/ionomycin-restimulated cells were fixed with 3% paraformaldehyde in PBS for 12 min followed by a 5-minute wash with PBS under centrifugation. Fixed cells were permeabilised with 0.1% saponin (Acros Organics) prepared in PBS and stained with IL-17-PE, IFNγ-e450, IL-21-APC, CD3-PERCP, CD4-FITC. For all studies cells were acquired on a Dako Cyan flow cytometer (Dako Cytomation) and data analysed using FlowJo software (Tree Star version 8.8.6). All antibodies were purchased from ebioscience/Thermofisher or BD Biosciences and expression quantified relative to the appropriate isotype control.

### 2.4 Quantitative real-time PCR

Total RNA was extracted by phenol/chloroform method after cell lysis in TRIzol (Life Technologies/Invitrogen). 0.3–0.5 μg RNA was reverse transcribed with random hexamers using TaqMan reverse transcription reagents (Thermofisher/Applied Biosystems). Quantitative real-time PCR for 18S rRNA, VDR, RXR, DRIP-205, NcOA1, NCOR1 and NCOR2, IL-17 or IFNγ was then performed on an Applied Biosystems 7900 machine using assays on demand from Applied Biosystems: 18S rRNA, (4319413E); VDR (Hs00172113_m1); RXR (Hs01067640_m1), NCoR1 (Hs01094540_m1), NCoR2 (Hs00196955_m1), DRIP205 (Hs01062349_m1), NCoA1 (Hs00186661_m1); IL-17 (Hs99999082_m1) and IFNγ(HS00989291_m1). Amplification of cDNAs involved incubation at 50 °C for 2 min and 95 °C for 10 min followed by 40 cycles of 95 °C for 15 s and 60 °C for 1 min. VDR mRNA expression was then calculated relative to 18S rRNA using the delta Ct method as described previously [30].

### 2.5 Statistical analysis

GraphPad Prism 5.0a software (GraphPad) was used for graphical summary and statistical analysis. For analysis of normality and Q-Q plot inspection SPSS statistics version 22 was used. Non-parametric Wilcoxon tests were used to test significance between two conditions. To test interactions between 1,25(OH)_2_D_3_ and cell source location repeated measures two factor within subject analysis with Huynf-Feldt correction was performed. The Shapiro-Wilk normality test and inspection of normal Q-Q plots were used to confirm that the data could be tested with these parametric models. For data sets that did not pass the normality test (VDR mRNA) the data were log_10_ transformed, since by this transformation the residuals from the mean became normally distributed.

## 3 Results

### 3.1 Anti-inflammatory effects of 1,25(OH)_2_D_3_ are reduced in synovial fluid T cells

**Fig. 1:**
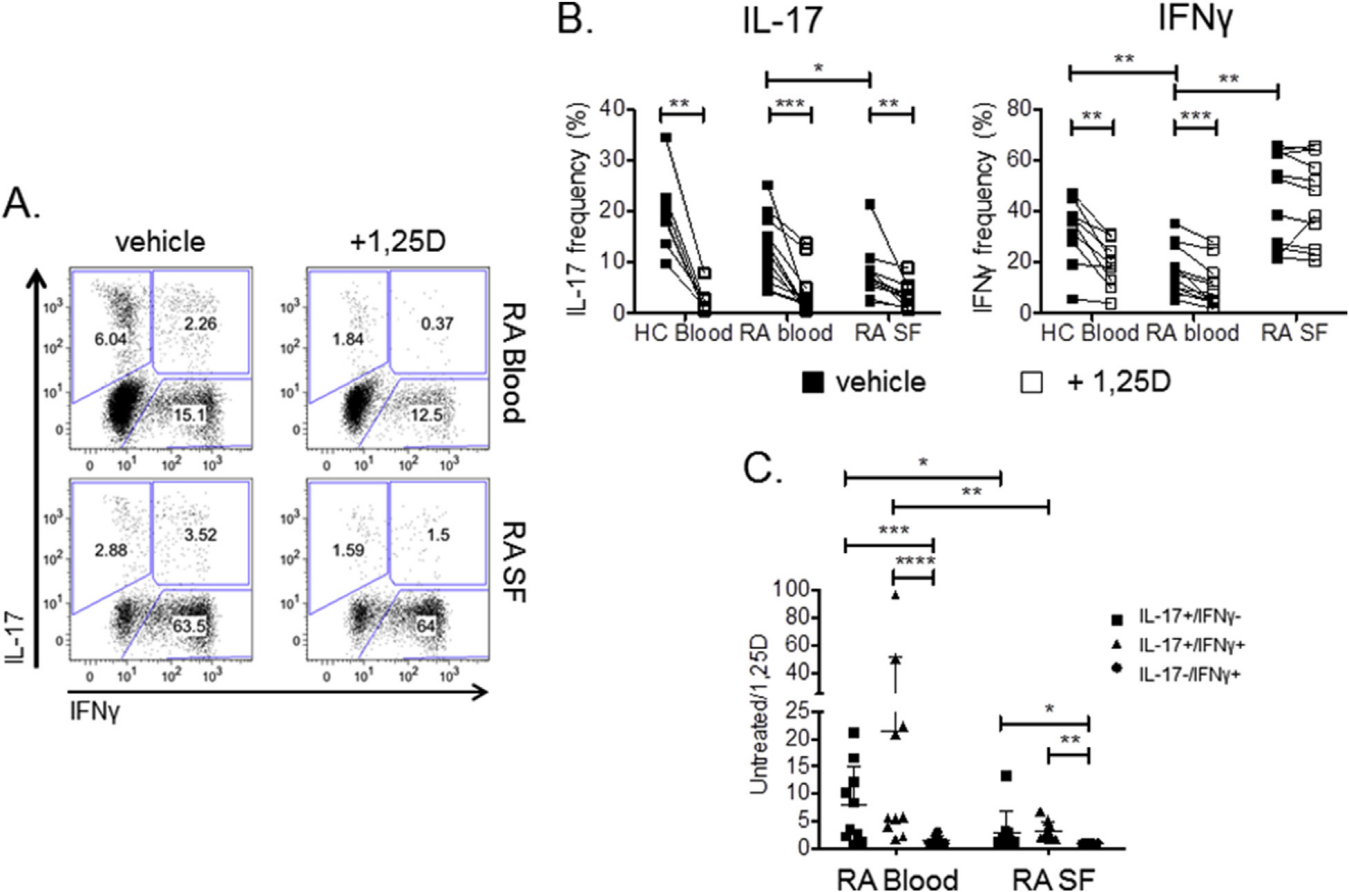
**Anti-inflammatory effects of 1,25(OH)**_**2**_**D**_**3**_**are reduced in T cells from synovial fluid compared to peripheral blood**. Mononuclear cells from the peripheral blood of healthy controls (HC) and the blood and synovial fluid (SF) of RA patients were stimulated with anti-CD3 for seven days in the presence or absence of 1,25(OH)_2_D_3_ (+1,25D) and frequencies of IL-17 + and IFNγ+ CD4^+^ T cells quantified by flow cytometry (**A**). Representative bivariate plots of IL-17 versus IFNγ expression by CD4^+^ T cells showing IL-17+/IFNγ- (Th17), IL-17+IFNγ+ (Th17.1) and IL-17-IFNγ+ (Th1) subsets for cells from RA blood vs RA SF. (**B**). Significant effects of 1,25(OH)_2_D_3_ were tested for HC (n = 9), RA blood (n = 12), RA SF (n = 11) by Wilcoxon matched pairs or Mann Whitney nonparametric tests as appropriate (**C**). Effect of 1,25(OH)_2_D_3_ on Th17, Th17.1 and Th1 populations quantified by frequency in respective gate under untreated/1,25D conditions. Significance was tested by repeated measures two-way ANOVA. ns = non-significant, * = P < 0.05, ** = P < 0.01, *** = P < 0.001.

To determine whether T cells from patients with active RA are sensitive to the anti-inflammatory effects of 1,25(OH)_2_D_3_, peripheral blood mononuclear cells (PBMC) from the blood of healthy controls and established RA patients, as well as synovial fluid (SF) mononuclear cells (SFMC) from RA patients, were stimulated with anti-CD3 in the presence or absence of 1,25(OH)_2_D_3_ for seven days and the frequency of CD4^+^ T cells expressing IL-17 or IFNγ measured by flow cytometry (Fig. 1A and B). 1,25(OH)_2_D_3_ decreased IL-17 + and IFNγ+ T cell frequencies in HC and RA PBMCs. It also reduced frequencies of IL-17 + T cells in SFMC cultures. However 1,25(OH)_2_D_3_ had no effect on the frequency of IFNγ+ T cells in SFMCs (Fig. 1B). Further classifying CD4^+^ T cells according to their combined expression of IL-17 and IFNγ revealed significantly greater suppressive effects of 1,25(OH)_2_D_3_ on Th17 (IL-17+IFNγ-) and Th17.1 (IL-17+IFNγ+) T cells in RA blood relative to SF, whilst Th1 (IL-17-IFNγ+) frequencies were not affected by 1,25(OH)_2_D_3_ in blood or SF (Fig. 1C). Thus Th17 and Th17.1 populations from the inflamed joints of RA patients are less sensitive to the anti-inflammatory effects of 1,25(OH)_2_D_3_ than equivalent circulating T cells. Similar results were also obtained with CD8^+^ T cells (Fig. S1), where 1,25(OH)_2_D_3_ decreased IL-17 + CD8^+^ frequencies in HC and RA PBMC, as well as SFMC. However, 1,25(OH)_2_D_3_ had no effect on the frequency of IFNγ+ CD8^+^ T cells in RA PBMC or SFMC.

### 3.2 Effects of 1,25(OH)_2_D_3_ are greater on blood naïve versus memory T cells

**Fig. 2:**
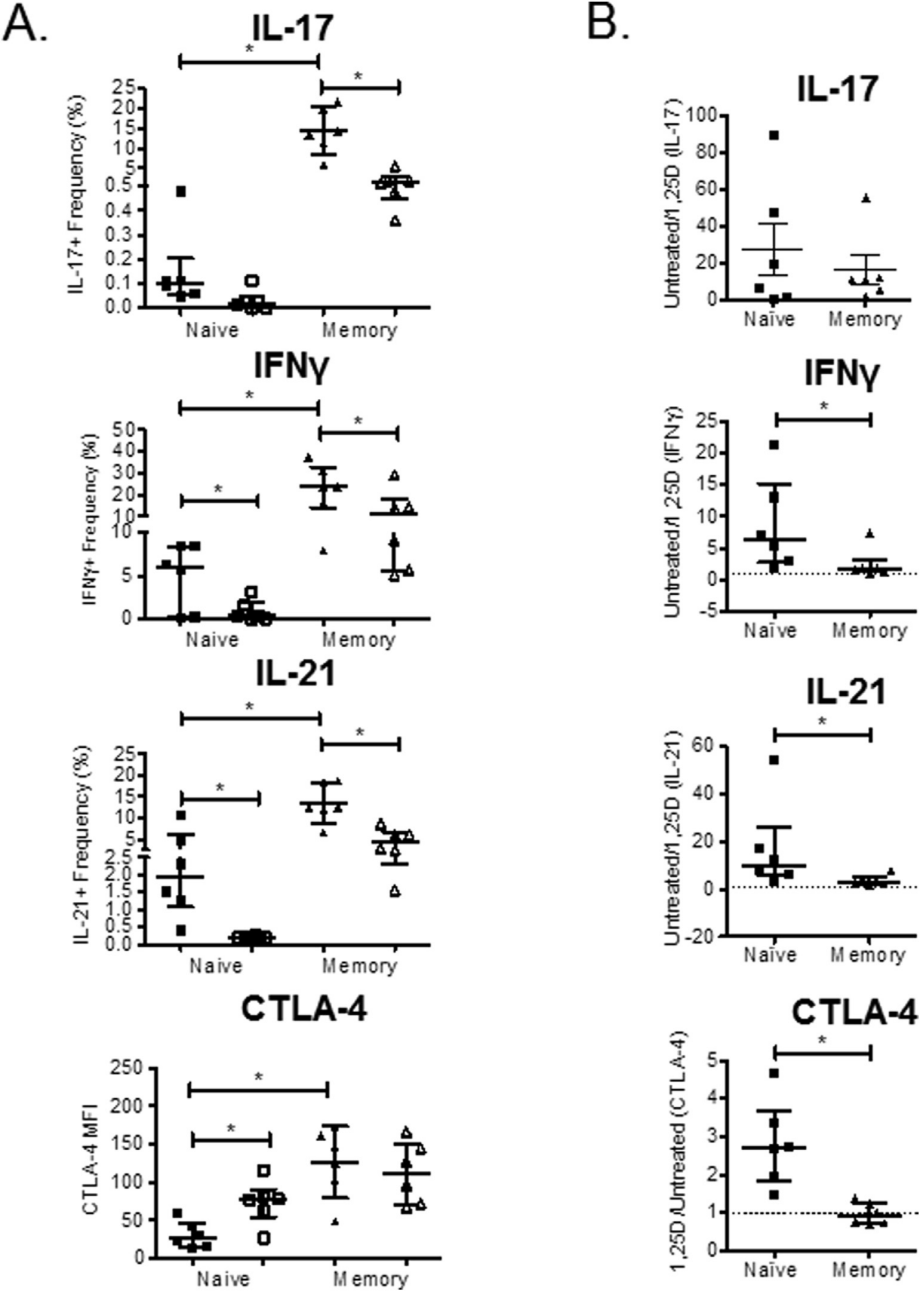
**Effects of 1,25(OH)**_**2**_**D**_**3**_**are greater on naïve than memory T cells**. Naive and memory CD4^+^ T cells were purified from the peripheral blood of healthy controls and stimulated with autologous monocytes and anti-CD3 in the presence or absence of 1,25(OH)_2_D_3_ (+1,25D). Frequency of cells expressing IL-17, IFNγ and IL-21 was quantified at seven days and expression of CTLA-4 and FoxP3 measured at four days by flow cytometry. (**A**) Frequencies of cytokine positive cells and CTLA-4 median fluorescence intensity (MFI) summarised for n = 6 donors for vehicle (closed symbols) and 1,25(OH)_2_D_3_ (+1,25D)-treated cells (open symbols). (**B**) The effect of 1,25(OH)_2_D_3_ on IL-17, IFNγ and IL-21 was compared for naïve and memory T cells by frequency of positive cells under untreated/1,25D conditions. A lower frequency limit of 0.001% was used for these calculations. 1,25(OH)_2_D_3_ effect on CTLA-4 was estimated by MFI under 1,25D/untreated conditions. Significant differences were tested using Wilcoxon matched pairs tests. ns = non-significant, * = P < 0.05, ** = P < 0.01, *** = P < 0.001.

Consistent with previous studies [31], only 47% (±12%) T cells isolated from blood were memory (CD45RO+). By contrast, 98% (±2%) SF T cells were CD45RO+ (Fig. S2). We therefore determined whether the decreased effect of 1,25(OH)_2_D_3_ upon SF T cells could be attributed to differences between memory and naïve T cells. As shown in Fig. 2A and Fig. S3, IL-17 + and IFNγ+ T cells were more abundant in memory T cell populations. Treatment with 1,25(OH)_2_D_3_ suppressed IFNγ+ and IL-17 + frequencies in both naïve and memory subsets. For IL-17, 1,25(OH)_2_D_3_ had equal effect on naïve and memory T cells whereas the effect of 1,25(OH)_2_D_3_ on IFNγ expression was greatest for naïve T cells (Fig. 2B). 1,25(OH)_2_D_3_ also had greatest effect upon IL-21 and CTLA-4 expression in naïve T cells compared to memory T cells, with 1,25(OH)_2_D_3_ inducing CTLA4 in naïve T cells but having no effect on memory T cells (Fig. 2B and Fig. S3).

As it is unclear how many rounds of stimulation circulating memory T cells have experienced *in vivo*, we also compared the effect of 1,25(OH)_2_D_3_ on naïve and memory T cells during sequential rounds of stimulation (Fig. S4). Analysis of three cytokines (IL-17, IFNγ, and IL-21) showed that 1,25(OH)_2_D_3_ was more effective in naïve versus memory T cells at the first round of stimulation, but this difference in sensitivity was lost following the second round of T cell stimulation. These data support the concept that induction of a memory T cell phenotype reduces the anti-inflammatory effects of 1,25(OH)_2_D_3_ on T cells, which may in part explain the differences in 1,25(OH)_2_D_3_ responses between blood and SF T cells.

### 3.3 1,25(OH)_2_D_3_ signaling components in naïve versus memory T cells

**Fig. 3:**
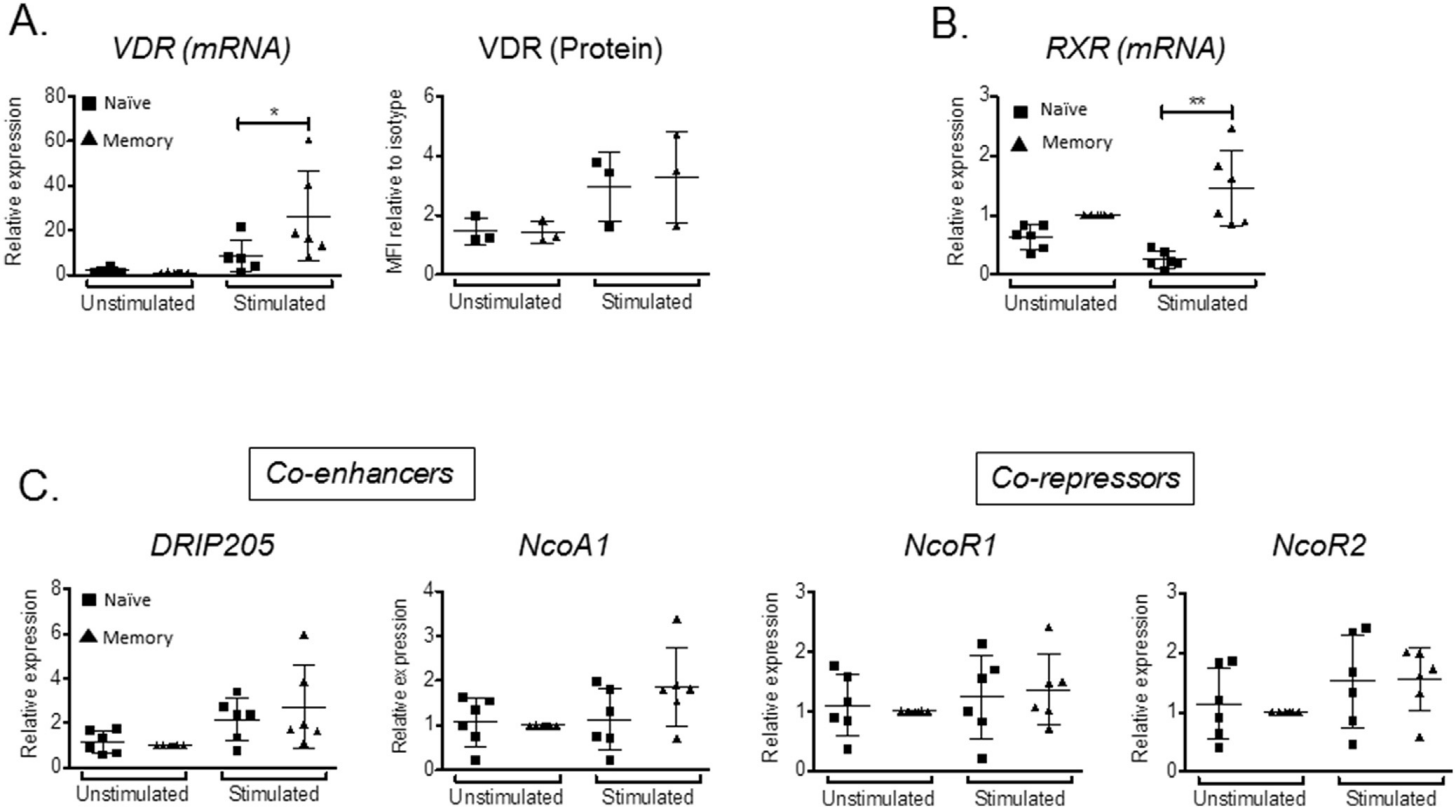
**Memory and naïve T cells express similar levels of vitamin D response machinery**. RNA was purified from naive and memory CD4^+^ T cells from the peripheral blood of healthy controls before and after stimulation with antiCD3/CD28 beads. Regulators of response to 1,25(OH)_2_D_3_ including **(A)** Vitamin D receptor (VDR), **(B)** Retinoid X Receptor (RXR), **(C)** VDR co-regulators: DRIP205, NcoA1, NcoR1 and NcoR2 were measured by qPCR. mRNA expression for each gene is shown relative to matched memory T cells. VDR protein in T cells was also measured by flow cytometry before and after stimulation with PMA and ionomycin. Stimulated T cells were defined as CD69^+^. Statistical significance was tested by repeated measures two-way ANOVA. * = P < 0.05, ** = P < 0.01.

To investigate the underlying basis for reduced 1,25(OH)_2_D_3_ responsiveness in memory versus naïve T cells, expression of the nuclear vitamin D receptor (VDR), its retinoid X receptor (RXR) heterodimer partner, as well as associated activator/repressor proteins was assessed. RT-PCR analyses and flow cytometry showed similar low levels of VDR in unstimulated naïve and memory T cells, with VDR expression increasing in both T cell populations following stimulation (p < 0.05 for mRNA and protein in stimulated naïve and memory T cells versus unstimulated naïve and memory T cells) (Fig. 3A). Similar to VDR, unstimulated naïve and memory T cells showed comparable low expression of RXR, but only memory T cells showed induction of this receptor upon stimulation (Fig. 3B). In addition to VDR and RXR, several co-regulators participate in 1,25(OH)_2_D_3_-mediated transcriptional responses, and these factors may contribute to variable responses to 1,25(OH)_2_D_3_ [32]. However, analysis of mRNAs for the co-enhancers DRIP205 and NcOA1, and co-repressors NcoR1 and NcoR2, showed no difference in expression between naïve and memory T cells (Fig. 3C). The decreased 1,25(OH)_2_D_3_ responsiveness of memory versus naïve T cells could therefore not be attributed to impaired expression of VDR or its signaling partners.

### 3.4 Effects of 1,25(OH)_2_D_3_ in SF versus blood memory T cells

**Fig. 4:**
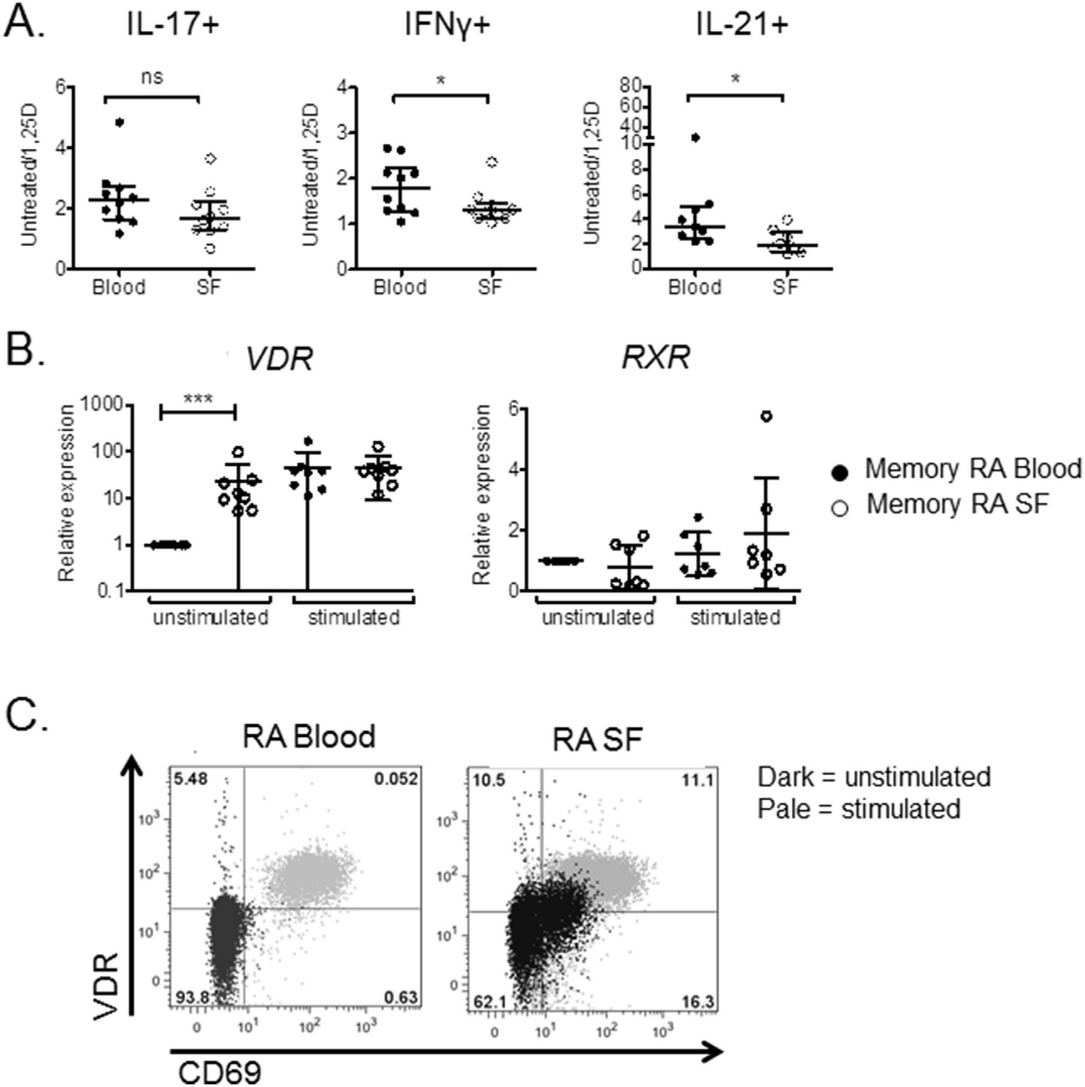
**Memory T cells from synovial fluid are relatively insensitive to 1,25(OH)**_**2**_**D**_**3**_**compared to memory T cells from peripheral blood**. CD45-RO + memory T cells were isolated from synovial fluid (SF) and peripheral blood of established RA patients and stimulated with anti-CD3/CD28 beads in the presence or absence of 1,25(OH)_2_D_3_ (+1,25D) overnight or for 5 days. Response to 1,25(OH)_2_D_3_ was assessed by measurement of IL-17, IFNγ and IL-21 by flow cytometry at 5 days and relative expression of cytokines in the presence vs the absence of 1,25(OH)_2_D_3_ calculated for n > 9 donors (**A**). Significant differences were tested using Wilcoxon matched pairs tests. ns = non-significant, * = P < 0.05. (**B**) Effects of cell location and 20hr stimulation on VDR and RXR mRNA transcripts were examined by qPCR (**B**). Expression is given relative to the level in unstimulated memory cells. Statistical significance was tested by repeated measures two-way ANOVA. * = P < 0.05, *** = P < 0.001, ns = not significant. VDR expression in CD4^+^ T cells from blood and synovial fluid was also assessed by flow cytometry for n = 3 donors. Representative data for CD69 versus VDR are shown in (**C)**.

To determine whether decreased responses to 1,25(OH)_2_D_3_ in SF compared to blood T cells involve differences between memory T cells from the two compartments, matched CD45RO + CD4^+^ memory T cells from paired blood and SF of RA patients were stimulated in the presence or absence of 1,25(OH)_2_D_3_. Flow cytometry for IFNγ and IL-21 showed decreased anti-inflammatory effects of 1,25(OH)_2_D_3_ in SF versus blood memory T cells, whilst a non-significant trend was observed for IL-17 (Fig. 4A). Flow cytometry and RT-PCR revealed enhanced VDR expression in unstimulated memory T cells isolated from SF relative to those obtained from blood (Fig. 4B and C), consistent with their more activated (CD69^+^) phenotype (Fig. 4C). VDR expression increased following memory T cell stimulation (p < 0.001 for stimulated versus unstimulated cells), but there was no significant difference in VDR expression between stimulated blood and stimulated SF cells (Fig. 4B). By contrast, mRNA for RXR did not differ in blood versus SF memory T cells in the stimulated or unstimulated state (Fig. 4B). The co-regulators DRIP205, NcoA1, NcoR1 and NcoR2 also showed elevated expression in unstimulated memory SF T cells (Fig. S5), similar to that observed for VDR. Collectively these data indicate that SF memory T cells are less affected by 1,25(OH)_2_D_3_ than memory T cells from blood, but this is not due to impaired expression of VDR or its signaling partners.

### 3.5 Effects of 1,25(OH)_2_D_3_ on Th17, Th17.1 and Th1 cells

**Fig. 5:**
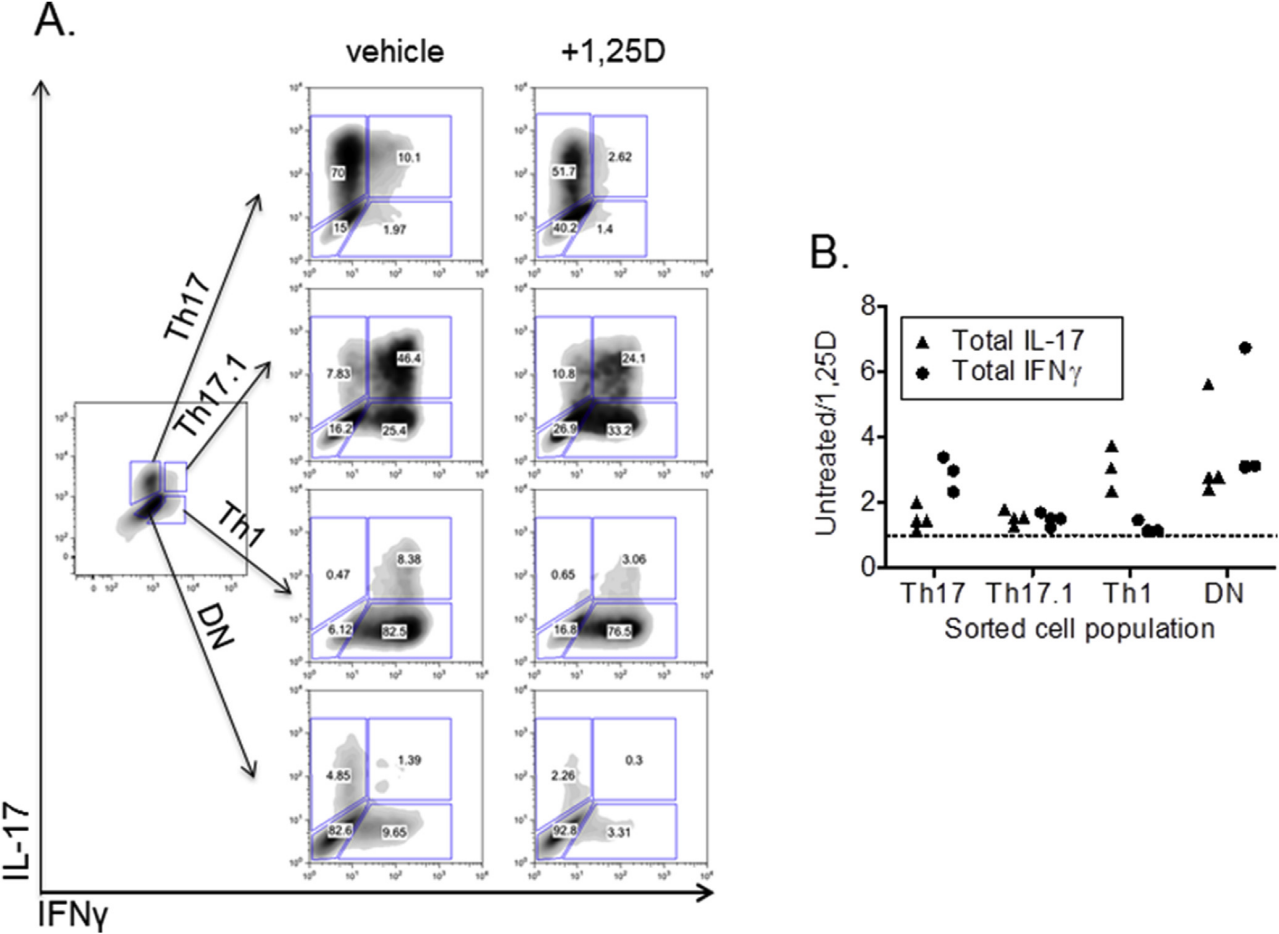
**1,25(OH)**_**2**_**D**_**3**_**suppresses cytokine expression by pre-committed Th17, Th17.1 and Th1 cells but most potently affects novel phenotype induction**. CD4^+^ T cells from healthy control blood were stimulated with monocytes and anti-CD3 for seven days and sorted into IL-17+IFNγ- (Th17), IL-17+IFNγ+ (Th17.1), IL-17-IFNγ+ (Th1) or double negative (DN) subsets using cytokine secretion assays and flow cytometry. After two day culture with monocytes and anti-CD3 in the presence or absence of 1,25(OH)_2_D_3_ (+1,25D) T cells were re-analysed for IL-17 and IFNγ expression by flow cytometry. (**A**) Data from one donor, representative of three studied. (**B**) Effect of 1,25(OH)_2_D_3_ upon total IL-17 and total IFNγ expression by sorted subsets, calculated as the ratio of cells expressing the cytokine under untreated vs treated conditions.

To date, reported anti-inflammatory effects of 1,25(OH)_2_D_3_ have been based on data from mixed cultures of cytokine-expressing and non-expressing T cells. Further studies were therefore carried out to determine the effects of 1,25(OH)_2_D_3_ on T cells expressing specific cytokines. By combining anti-IL-17-PE and anti-IFNy-APC cytokine detection reagents, live T cells were labeled according to their expression of IL-17 and/or IFNγ, and Th17, Th17.1, Th1 cells were obtained by FACS as well as T cells double negative for IL-17 and IFNγ (DN). RT-PCR analysis of IL-17 and IFNγ mRNA in the sorted populations confirmed cytokine purity of the sorted cells (Fig. S6). The Th17, Th17.1 and Th1 T cells generated in this manner were then re-stimulated in the presence or absence of 1,25(OH)_2_D_3_ for two days. In vehicle-treated re-stimulated populations, we observed plasticity of phenotype between Th17, Th17.1 and Th1, including the generation of T cells double negative for IL-17 and IFNγ (Fig. 5A). Th17 cells could give rise to IFNγ-expressing T cells, and Th1 cells could induce IL-17expression. DN cells could also give rise to IL-17 and IFNγ (Fig. 5A).

Treatment with 1,25(OH)_2_D_3_ reduced expression of IL-17 or IFNγ in T cells pre-committed to Th17 and Th1 phenotypes respectively, but 1,25(OH)_2_D_3_ had a much greater inhibitory effect on *de novo* expression of IL-17 or IFNγ (Fig. 5A and B). In Th17 cells, 1,25(OH)_2_D_3_-sensitivity was greater for IFNγ than IL-17, whilst the opposite response was observed in Th1 cells. Moreover, 1,25(OH)_2_D_3_ actively suppressed the development of Th17, Th17.1 and Th1 populations from DN T cells, but had little effect on IL-17 or IFNγ expression in Th17.1 T cells that had pre-existing expression of both IL-17 and IFNγ (Fig. 5B).

### 3.6 Capacity for phenotype change is low in SF T cells

**Fig. 6:**
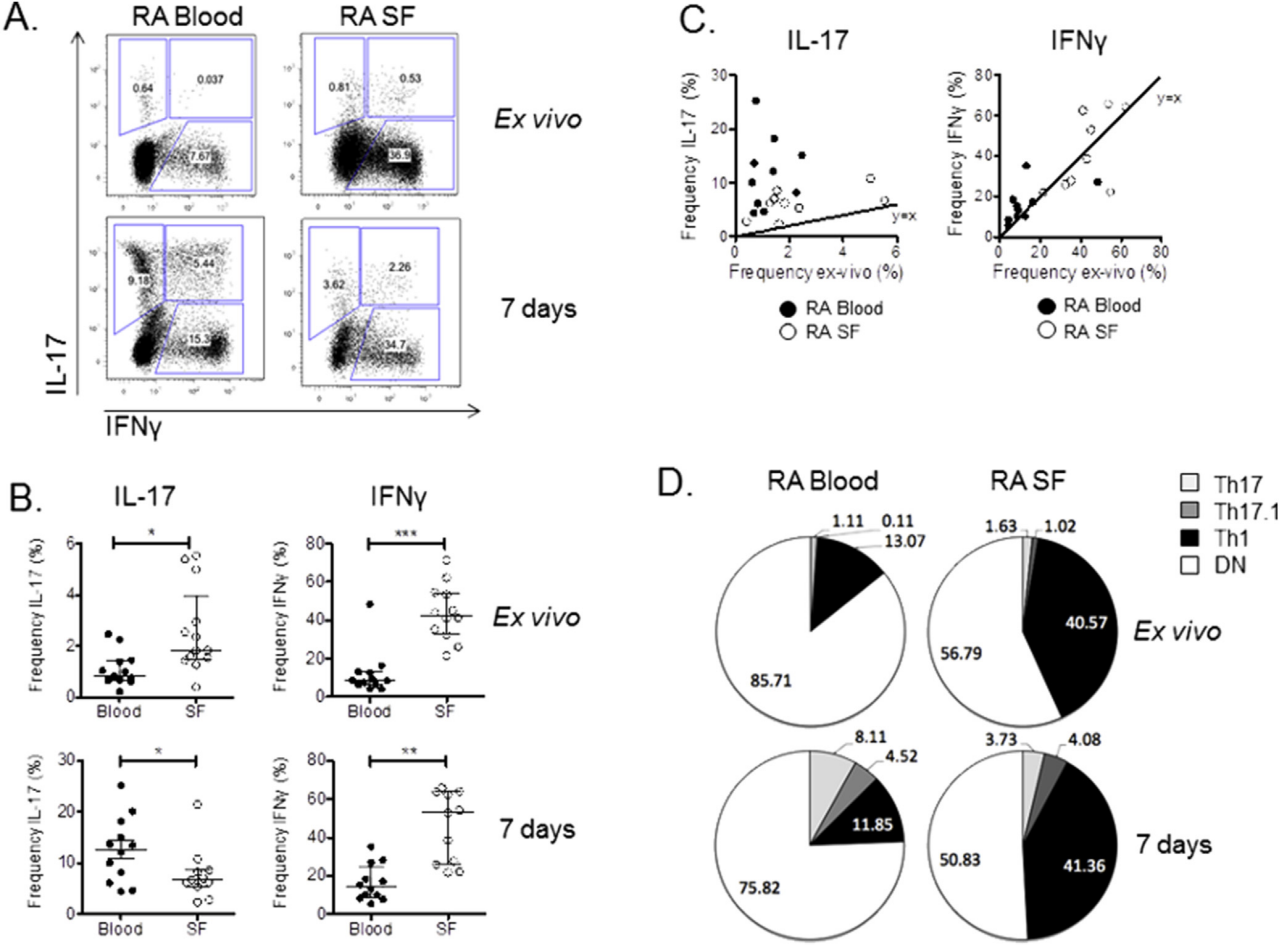
**Capacity for phenotype change upon stimulation is reduced in SF T cells**. IL-17 and IFNγ expression by CD4^+^ T cells from the peripheral blood and synovial fluid of RA patients was measured by flow cytometry *ex vivo* and after anti-CD3 stimulation for seven days. **(A)** Representative bidirectional FACS plots of IL-17 versus IFNγ in T cells from blood and synovial fluid (SF) from a single RA donor *ex vivo* and after 7 days of culture. (**B**) Summary of T cell frequencies in blood and synovial fluid (SF) for multiple donors *ex vivo* and after 7 days of culture. Significant differences were tested using Wilcoxon matched pairs tests. * = P < 0.05, ** = P < 0.01, *** = P < 0.001. **(C)** XY plots for frequencies of IL-17 + and IFNγ+ CD4^+^ T cells *ex vivo* and after seven days. Diagonal lines indicate equal frequencies at each time point. **(D)** Pie charts summarizing the frequencies of Th17 (pale grey), Th17.1 (dark grey), Th1 (black) and double negative (DN) (white) CD4^+^ T cells from blood and SF *ex vivo* and after 7 days of culture (n = 12 donors).

Further studies were carried out to determine if the impaired response to 1,25(OH)_2_D_3_ observed for SF versus blood T cells was due to differences in T cell phenotype commitment and plasticity. This was carried out by comparing the cytokine profiles of blood and SF T cells *ex vivo* and after stimulation. There were few IL-17 + T cells in blood *ex vivo* and these were almost exclusively Th17 (Fig. 6A, B and D). IL-17 expression was also low in SF, but this was evenly distributed across Th17 and Th17.1 populations (Fig. 6D). Compared to IL-17, considerably more T cells expressed IFNγ *ex vivo* in blood and SF, however, the frequency of IFNγ+ cells was much higher in SF compared to paired blood. Culture of T cells from blood for 7 days resulted in significant expansion of IL-17 + T cells (12-fold) (Fig. 6C), with the Th17.1 population showing greatest fold change (50-fold) (Fig. 6D). IFNγ+ Th1 cells from blood showed no change in frequency from *ex vivo* to 7 days of culture (Fig. 6A and D). In contrast to blood, culture of T cells from SF resulted in only a 2-fold induction of Th17 T cells and a 4-fold induction of Th17.1 T cells, with Th1 cells (the largest fraction of IFNγ+ T cells in SF), again showing no change in frequency from *ex vivo* to 7 days of culture (Fig. 6D).

**Fig. 7:**
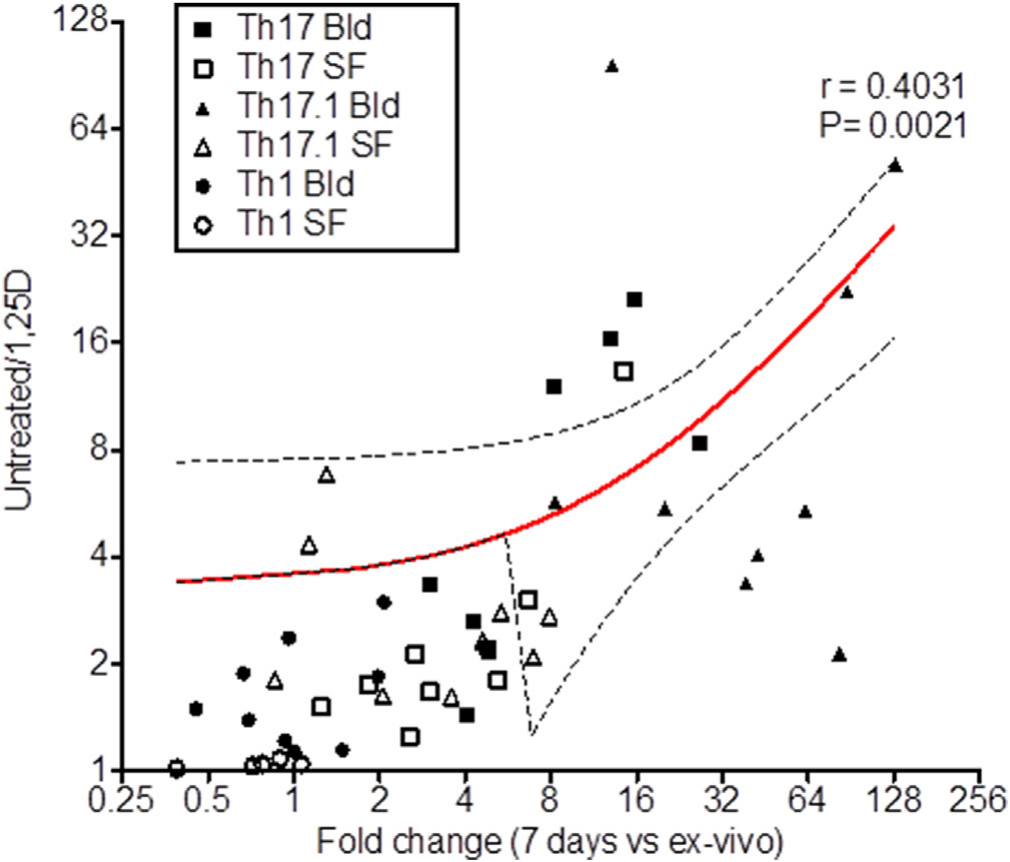
**T cell responses to 1,25(OH)**_**2**_**D**_**3**_**correlate directly with capacity for phenotype change**. Bidirectional plot of fold change in frequency of the population (Th17 blood (Bld), Th17 synovial fluid (SF), Th17.1 Bld, Th17.1 SF, Th1 Bld, Th1 SF) after seven day culture under control conditions versus the ratio of the population frequency in the presence versus the absence of 1,25(OH)_2_D_3_. Correlation significance was tested by Pearson's analysis and the linear regression line with 95% confidence interval (hashed line) is shown.

The capacity for expansion of IL-17 + T cells during culture correlated directly with the ability of these cells to respond to 1,25(OH)_2_D_3_, with Th17 and Th17.1 cells from blood being more effectively suppressed by 1,25(OH)_2_D_3_ than their SF counterparts (Fig. 7). By contrast, the lack of phenotype plasticity shown for Th1 cells and cells from SF (Fig. 6A–D), was associated with lower sensitivity to 1,25(OH)_2_D_3_ (Fig. 7). The lack of an anti-inflammatory response to 1,25(OH)_2_D_3_ in SF T cells therefore appears to be due to the limited phenotype plasticity exhibited by these cells.

## 4 Discussion

Vitamin D metabolites such as pro-hormone 25-hydroxyvitamin D (25OHD) and active 1,25(OH)_2_D_3_ are detectable in SF from RA patients [19], [33], and monocytes isolated from SF have been shown to actively synthesize 1,25(OH)_2_D_3_ from 25OHD [19], [34]. Thus, there is significant potential for elevated concentrations of 1,25(OH)_2_D_3_ within inflamed joints. In addition, VDR is broadly expressed and present within cells from synovial tissue and fluid [35] indicating that different components of the inflamed joint have the capacity to respond to 1,25(OH)_2_D_3_, in an intracrine or paracrine fashion [18]. As recently summarized [18], multiple *in vitro* studies also demonstrate anti-inflammatory effects of 1,25(OH)_2_D_3_ upon the immune cells that contribute to RA pathology. However, these studies have focused on cells from the blood of healthy controls or RA patients. The extent to which anti-inflammatory responses to 1.25(OH)_2_D_3_ also occur in immune cells from inflamed tissue is still poorly understood, but is nevertheless crucially important when considering the potential use of 1,25(OH)_2_D_3_ as a therapy for inflammatory diseases.

Th1 and Th17 cells are believed to contribute to inflammatory conditions such as RA [36], and are both recognized inhibitory targets for 1,25(OH)_2_D_3_. Vitamin D supplementation may prevent, slow or reverse inflammatory disease by inhibiting these T cell subsets. The current study addressed the question of 1,25(OH)_2_D_3_ responsiveness in T cells from inflamed tissues.

Initial experiments (Fig. 1) utilized PBMC and SFMC cultures that included mixtures of cells including different types of T cells and antigen-presenting cells such as dendritic cells (DC). In this setting, and *in vivo*, the overall effect of 1,25(OH)_2_D_3_ on T cells may involve indirect pathways of immunosuppression. For example, indirect suppression of IL-17/IFNγ effector T cell frequency by 1,25(OH)_2_D_3_ may occur via promotion of regulatory T cell (Treg) development [37]. In supplementary studies FoxP3+CD25 ^+^ Treg were found to be more abundant *ex vivo* in SFMC relative to PBMCs (Fig. S7A) but there was no difference between the frequency of FoxP3+CD25 ^+^ cells in PBMC and SFMC cultures after CD25 stimulation (Fig. S7B). Interestingly, 1,25(OH)_2_D_3_ increased expression of CD25 in both PBMC and SFMC and CTLA-4 in SFMC alone (Fig. S7B). However, in contrast to PBMC, 1,25(OH)_2_D_3_ did not increase FoxP3+CD25 ^+^ T cell frequencies in SFMC. It is therefore possible that the suppressive effect of 1,25(OH)_2_D_3_ upon CD4^+^ T cell IL-17 and IFNγ in PBMC cultures relative to SFMC involves, in part, enhancement of Treg differentiation and therefore stronger 1,25(OH)_2_D_3_-mediated effector T cell immunosuppression PBMC relative to SFMC.

1,25(OH)_2_D_3_ also has profound effects on antigen presenting cells (APC) such as dendritic cells (DC) that may indirectly impact T cell function [38]. It is also important to recognize that APC are the main source of active 1,25(OH)_2_D_3_ from precursor 25(OH)D_3_ for T cells *in vivo* [5], [6]. Thus, the potential contribution of DC to altered 1,25(OH)_2_D_3_ sensitivity of T cells from synovial fluid is likely to be highly complex and warrants detailed future study that considers responses to 1,25(OH)_2_D_3_ and 25(OH)D_3_. By isolating T cells from SFMC and PBMC we were able to clearly demonstrate that the lack of anti-inflammatory response to 1,25(OH)_2_D_3_ in mixed cell cultures of SFMC involves, at least in part, an intrinsic insensitivity to 1,25(OH)_2_D_3_ by CD4^+^ T cells at the inflamed site. We therefore predict that similar T cell insensitivity to 1,25(OH)_2_D_3_ occurs *in vivo* within the inflamed joints of RA patients. Conversely, the lack of effect of 1,25(OH)_2_D_3_ on IFNγ production by CD8^+^ T cells even in PBMC may reflect the fact that the IL-17+IFNγ+ population of IFNγ+ cells that is the principal target for 1,25(OH)_2_D_3_ is minimal in CD8^+^ T cells relative to CD4^+^ T cells, even under control conditions (Fig. S1).

Both naïve (CD45RA+) and memory (CD45RO+) T cells were able to respond to 1,25(OH)_2_D_3_, but inhibition of inflammatory cytokines was more pronounced for naïve T cells, and became less effective upon transition from naïve to memory. These findings suggest that variations in the proportion of naïve to memory T cells play a key role in defining the anti-inflammatory impact of 1,25(OH)_2_D_3_ *in vivo*, especially at the inflammatory site where memory T cells are the dominant T cell type [31]. Furthermore, whilst naïve to memory transition accounted for most of the loss in 1,25(OH)_2_D_3_ effect upon SF T cells, comparing memory T cells from blood with those from SF revealed a further decline in 1,25(OH)_2_D_3_ sensitivity in SF cells. Collectively, these data indicate that analysis of 1,25(OH)_2_D_3_-responses using T cells from blood, even blood from RA patients, may over-estimate the ability of vitamin D to regulate inflammatory T cells in disease-affected synovial fluid.

Th17 and Th1 T cell subsets not only play a critical role in the pathophysiology of inflammatory diseases such as RA, they are also pivotal to host responses to infectious pathogens such as *Mycobacterium tuberculosis* [39]. In this setting, 1,25(OH)_2_D_3_ is proposed to be less effective in the early stages of infection, where T cells are not chronically activated and therefore have lower levels of VDR expression. The resulting insensitivity to 1,25(OH)_2_D_3_ during early infection is advantageous in enabling maintenance of inflammatory T cell populations for maximal clearing of pathogenic antigen [40]. By contrast, at later stages of immune response to infection, more activated T cells express higher levels of VDR and are therefore more sensitive to 1,25(OH)_2_D_3_, with the principal response being minimization of T cell activity and chronic inflammation [40]. Data from the current study suggest that in chronically activated T cells from inflammatory sites such as the rheumatoid joint, there is re-instigation of 1,25(OH)_2_D_3_-insensitivity. However, in contrast to early stage T cell responses, decreased responsiveness to 1,25(OH)_2_D_3_ in activated SF T cells does not appear to be due to lower VDR-binding capacity for 1,25(OH)_2_D_3_: VDR expression was increased in SF T cells *ex vivo* consistent with the activated CD69 ^+^ phenotype of these cells, and levels of VDR and co-regulator proteins were similar for blood and SF T cells following *in vitro* stimulation. Likewise differential expression of VDR and its co-regulators did not account for the lesser effect of 1,25(OH)_2_D_3_ on anti-inflammatory responses by memory vs naïve T cells.

Several single nucleotide polymorphisms (SNPs) in the *VDR* gene have been associated with RA risk and severity [41], although the functional consequence of these *VDR* genetic variations yet to be defined. *VDR* polymorphisms were not explored in this study. Whilst it is possible that differences could influence patient sensitivity to 1,25(OH)_2_D_3_ systemically, it is unlikely that they would contribute to the differential 1,25(OH)_2_D_3_ sensitivity of blood versus SF T cells as the same *VDR* would be expressed in different tissues. In addition to SNPs, however, a number of 5′ variant *VDR* transcripts have been described that are driven by alternative promoters and display tissue specific patterns [42]. It is therefore possible that an inflammation-induced switch in VDR isoform could influence the sensitivity of SF T cells relative to blood despite equivalent levels of VDR expression, and this will be a key target for future studies of 1,25(OH)_2_D_3_ sensitivity in RA.

An additional feature of memory T cells versus naïve is their commitment to phenotype as defined by cytokine expression. T cells that express multiple inflammatory cytokines including those doubly expressing IL-17 and IFNγ (Th17.1 cells) are regarded the most pathogenic in inflammatory disease [43]. Correspondingly, Th17.1 cells were present at increased frequency in SF T cells *ex vivo* compared to blood. Previous studies into 1,25(OH)_2_D_3_ effects upon T cell cytokine expression have used mixed cultures of cytokine-expressing and non-expressing cells, making it impossible to determine effects of 1,25(OH)_2_D_3_ upon phenotype-committed T cells which, as our data on phenotype maintenance following stimulation suggest, are more enriched in SF compared to blood. By using novel cytokine cell capture methods to sort live Th17, Th17.1, Th1 and double negative cells into separate populations, the current study showed that responses to 1,25(OH)_2_D_3_ are in fact highly dependent on the commitment of T cells to a particular phenotype: 1,25(OH)_2_D_3_ treatment strongly suppressed conversion of cytokine naïve T cells into inflammatory effector cells as well as the transition of one effector phenotype to another through induction of a new cytokine. By contrast, 1,25(OH)_2_D_3_ was much less efficient at suppressing an existing T cell phenotype. T cells from SF displayed a more committed phenotype relative to blood as indicated by only small increases in Th17 and Th17.1 and no change in Th1 frequency following stimulation. Thus, whilst 1,25(OH)_2_D_3_ reduced expression of IL-17 and IFNγ in less phenotype-committed blood T cells by preventing new cytokine gene expression, it was much less effective as an inhibitor of established cytokine gene expression in SF T cells.

An important conclusion from these studies is that terminal commitment of memory T cells to a specific phenotype, as prevalent in disease-affected tissues such as RA synovium, plays a central role in attenuating anti-inflammatory effects of 1,25(OH)_2_D_3_. In concert with 1,25(OH)_2_D_3_, VDR functions as a transcription factor to regulate expression of genes such as IL-17 and IFNγ that have functional vitamin D response elements (VDRE) [44], [45]. This process is subject to genomic variation, but is also strongly influenced by epigenetic mechanisms that may lead to intra-individual [46], or disease-specific [32] changes in 1,25(OH)_2_D_3_-VDR signaling. Recent studies have shown that memory T cell development is associated with specific epigenetic signatures that strongly influence transcriptional regulators [47]. Thus, it seems likely that variations in response to 1,25(OH)_2_D_3_ according to naïve and memory state and extent of phenotype commitment will be due to epigenetic modifications that could strongly influence chromatin remodeling, and VDRE accessibility. In future studies it will be interesting to investigate the potential use of epigenetic modulators in correcting the 1,25(OH)_2_D_3_-insensitivity characteristic of T cells from RA disease-associated tissue.

## 5 Conclusion

Data in the current study endorse the potential use of vitamin D and/or 1,25(OH)_2_D_3_ in controlling the *de novo* generation of Th17 and Th17.1 cells that are associated with inflammatory disease. However, the limited effect of 1,25(OH)_2_D_3_ on phenotype-committed T cells from the site of inflammation suggests that vitamin D supplementation is unlikely to be successful as treatment for established active RA patients. Consistent with this hypothesis, not all early vitamin D supplementation trials in RA have reported positive effects of the treatment [48], [49], [50]. The fact that peripheral blood T cells from patients were responsive to the anti-inflammatory effects of 1,25(OH)_2_D_3_, and equally as responsive as those from healthy donors, nevertheless supports a use for vitamin D in the management of RA, either as a prophylactic or to reduce the risk of future disease flares in patients in remission. Owing to its powerful anti-inflammatory effects *in vitro*, vitamin D supplementation has been proposed as a cost-efficient, ‘natural’ and low-risk treatment strategy in multiple inflammatory diseases. However, since memory T cells with committed inflammatory phenotypes dominate the inflamed tissues in multiple inflammatory conditions, this study raises questions about widespread application of vitamin D therapy in the management of inflammatory diseases, especially cases of established, active disease. Further studies are required to identify the cause of the insensitivity to 1,25(OH)_2_D_3_ at the molecular level. Strategies to overcome this and restore 1,25(OH)_2_D_3_ sensitivity in inflammatory phenotype-committed memory T cells at the inflamed site may provide a novel approach to the treatment of RA and other inflammatory diseases.

## Appendix A Supplementary data

mmc1 mmc1
