# Supplementary material for: Decreased sensitivity to 1,25-dihydroxyvitamin D3 in T cells from the rheumatoid joint
Source: J Autoimmun. 2018 Mar;88:50–60. doi: 10.1016/j.jaut.2017.10.001 (PMC5854374; doi:10.1016/j.jaut.2017.10.001)
Supplement: mmc1 [file mmc1.pdf]

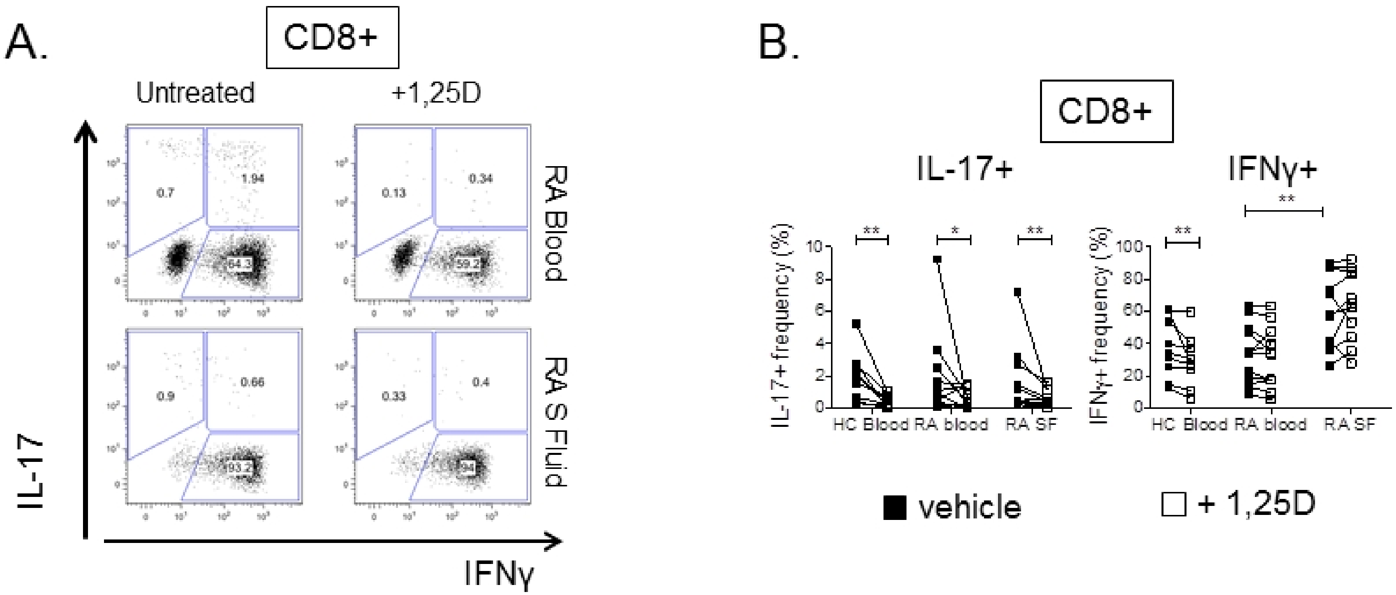

S1. Anti-inflammatory effects of 1,25(OH)$_2$D$_3$ are reduced in CD8+ T cells from RA patients. (**A**). Representative bivariate plots of IL-17 versus IFNγ expression by CD8+ T cells. (**B**). Significant effects of 1,25(OH)$_2$D$_3$ were tested for HC (n=9), RA blood (n=12), RA SF (n= 11) by Wilcoxon matched pairs or Mann Whitney nonparametric tests as appropriate. ns= non-significant, * = P<0.05, **=P<0.01, ***=P<0.001.

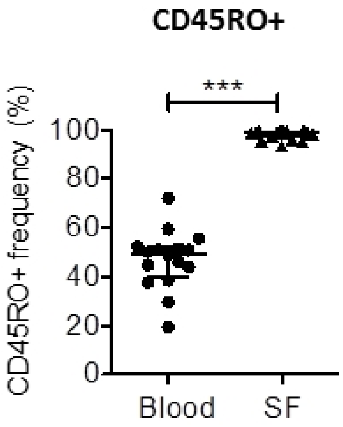

S2. Memory T cells are abundant in synovial fluid. Frequency of CD45RO+ CD4+ T cells in the blood and synovial fluid (SF) of RA patients was assessed by flow cytometry. Significance was tested by Wilcoxon matched pairs analysis *** = P<0.001.

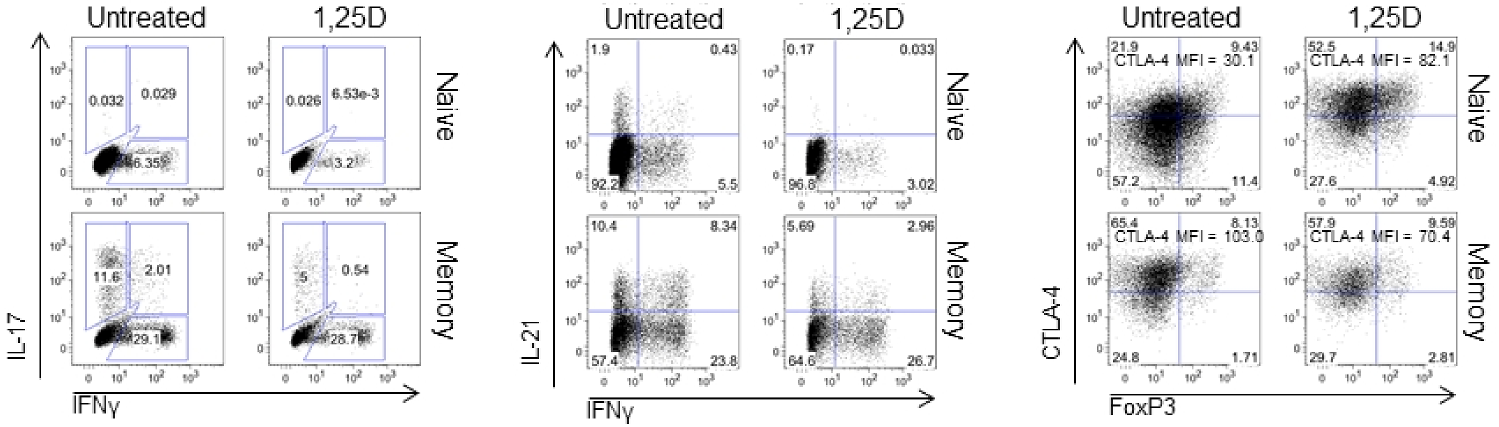

S3. Effects of 1,25(OH)$_2$D$_3$ are greater on naïve than memory T cells. Naive and memory CD4+ T cells were purified from the peripheral blood of healthy controls and stimulated with autologous monocytes and anti-CD3 in the presence or absence of 1,25(OH)$_2$D$_3$ (+1,25D). Expression of inflammatory cytokines IL-17, IFNγ and IL-21 was quantified at seven days and expression of CTLA-4 and FoxP3 measured at four days. Bivariate FACS profiles for one donor, representative of six are shown.

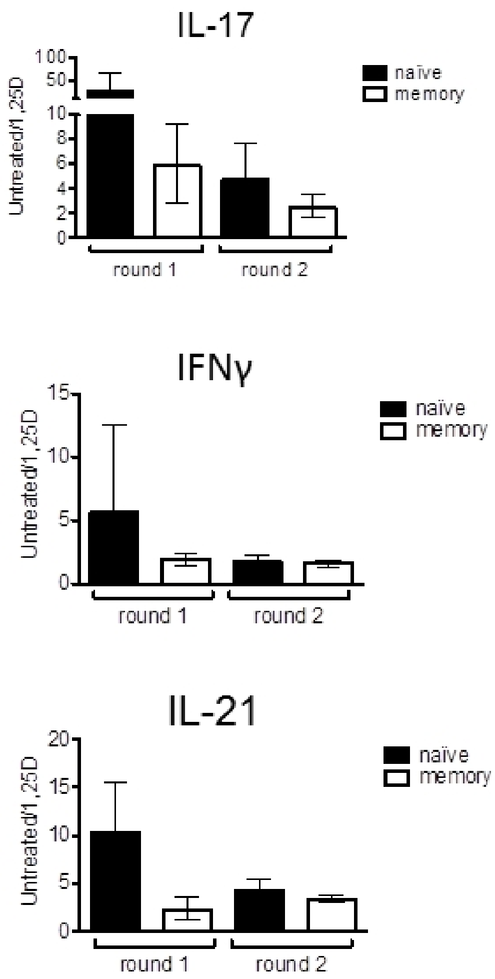

S4. Naïve T cell sensitivity to $1,25(OH)_2D_3$ decreases following stimulation

Naïve and memory CD4+ T cells were stimulated with monocytes and anti-CD3 for two sequential rounds of stimulation. The effect of $1,25(OH)_2D_3$ upon IL-17+, IFNγ+ and IL-21+ frequencies is given as the relative frequency of positive cells under untreated/$1,25(OH)_2D_3$ conditions. Graphs show the mean and range of effect for n=3 donors.

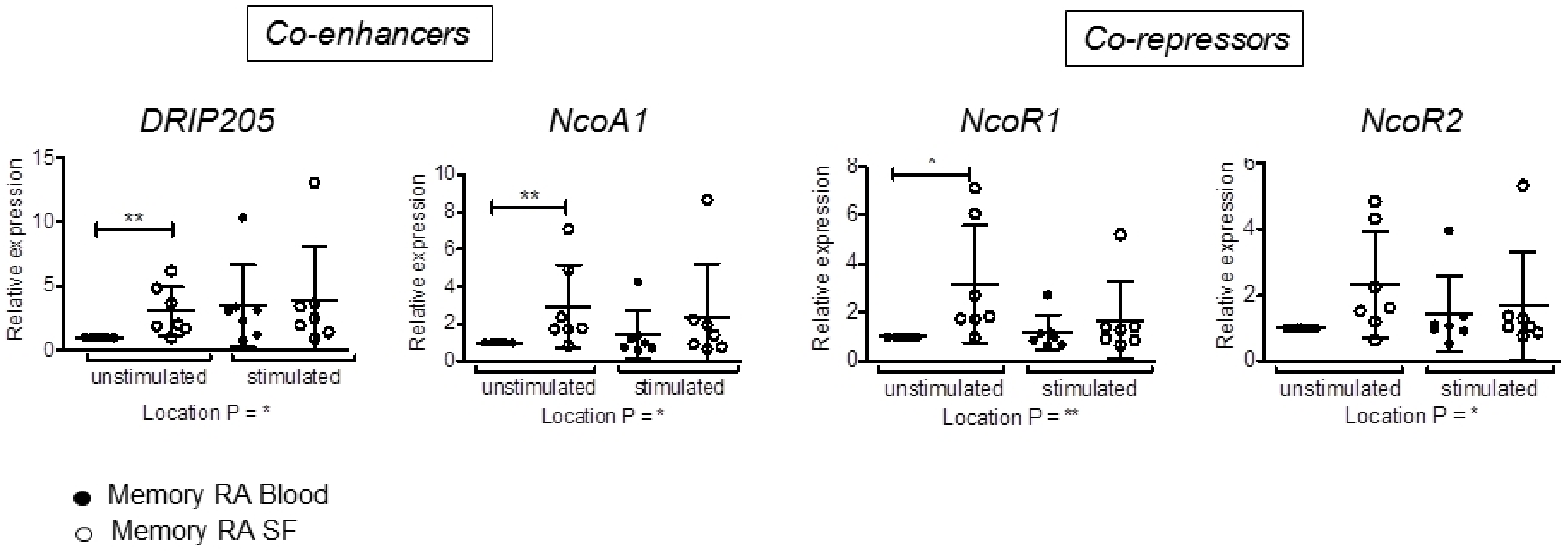

S5. Expression of mRNA for VDR co-regulators in blood versus SF memory T cells

In CD45RO+ memory T cells from RA patient blood and SF, qPCR was used to measure mRNA for the VDR co-activators DRIP205, NcoA1 and the VDR co-repressors NcoR1 and NcoR2. Data are shown for n=7 donors. Statistical significance was tested by repeated measures two-way ANOVA. * = P<0.05, **=P<0.01.

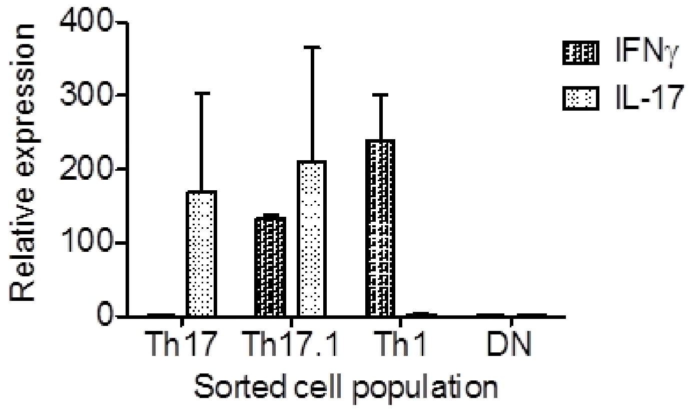

S6. IL-17 and IFNγ expression in purified Th17, Th17.1, Th1 and DN CD4+ T cells. qPCR was used to measure IL-17 and IFNγ mRNA expression in CD4+ T cells sorted into Th17, Th17.1, Th1 and DN subsets using cytokine secretion assays and flow sorting. Expression is given relative to the level in DN T cells.

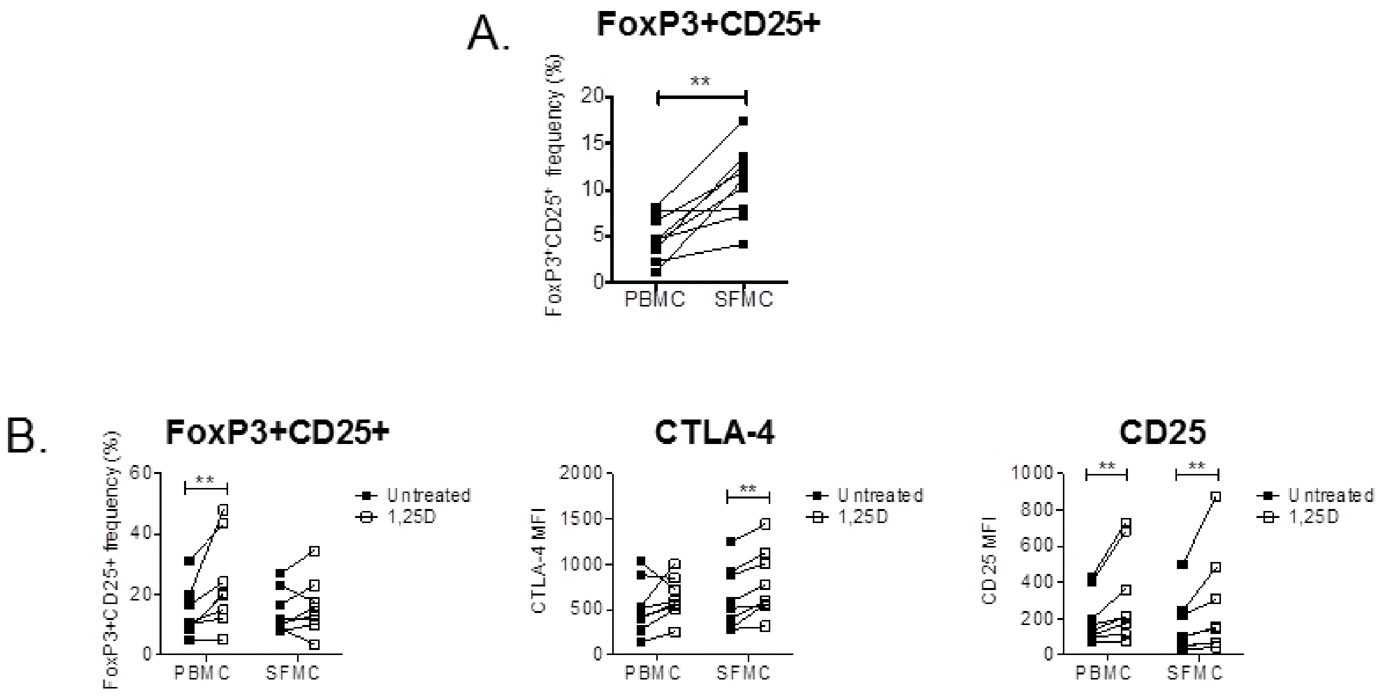

S7. 1,25(OH)$_2$D$_3$ regulation of Treg markers in PBMC and SFMC from RA patients. A) Ex-vivo frequencies of FoxP3+CD25+ CD4+ T cells. **B)** Frequencies of FoxP3+CD25+ CD4 T Cells and their median expression of CTLA-4 and CD25 in stimulated PBMC and SFMC cultures at three or four days. Significance was tested by Wilcoxon matched pairs analysis ** = P<0.01.
